# Phase I study to determine the safety, tolerability and immunostimulatory activity of thalidomide analogue CC-5013 in patients with metastatic malignant melanoma and other advanced cancers

**DOI:** 10.1038/sj.bjc.6601579

**Published:** 2004-03-02

**Authors:** J B Bartlett, A Michael, I A Clarke, K Dredge, S Nicholson, H Kristeleit, A Polychronis, H Pandha, G W Muller, D I Stirling, J Zeldis, A G Dalgleish

**Affiliations:** 1 Division of Oncology, Department of Cellular & Molecular Medicine, St George's Hospital Medical School, Cranmer Terrace, Tooting, London SW17 ORE, UK; 2 Celgene Corporation, Warren, NJ, USA

**Keywords:** phase I, CC-5013, thalidomide, immunomodulation, cytokines, T-cell activation

## Abstract

We assessed the safety, tolerability and efficacy of the immunomodulatory drug, CC-5013 (REVIMID™), in the treatment of patients with metastatic malignant melanoma and other advanced cancers. A total of 20 heavily pretreated patients received a dose-escalating regimen of oral CC-5013. Maximal tolerated dose, toxicity and clinical responses were evaluated and analysis of peripheral T-cell surface markers and serum for cytokines and proangiogenic factors were performed. CC-5013 was well tolerated. In all, 87% of adverse effects were classified as grade 1 or grade 2 according to Common Toxicity Criteria and there were no serious adverse events attributable to CC-5013 treatment. Six patients failed to complete the study, three because of disease progression, two withdrew consent and one was entered inappropriately and withdrawn from the study. The remaining 14 patients completed treatment without dose reduction, with one patient achieving partial remission. Evidence of T-cell activation was indicated by significantly increased serum levels of sIL-2 receptor, granulocyte–macrophage colony-stimulating factor, interleukin-12 (IL-12), tumour necrosis factor-*α* and IL-8 in nine patients from whom serum was available. However, levels of proangiogenic factors vascular endothelial growth factor and basic foetal growth factor were not consistently affected. This study demonstrates the safety, tolerability and suggests the clinical activity of CC-5013 in the treatment of refractory malignant melanoma. Furthermore, this is the first report demonstrating T-cell stimulatory activity of this class of compound in patients with advanced cancer.

CC-5013 is in development for the treatment of a variety of oncological and inflammatory diseases. The first available immunomodulatory drug (IMiD), Thalomid® (thalidomide), is approved for the treatment of the cutaneous manifestations of moderate to severe erythema nodosum leprosum (ENL). Thalidomide has also been shown to be a useful drug in a wide range of other clinical conditions for which there is little other treatment option (Marriott *et al*, 1999; Raje and Anderson, 1999). These include rheumatoid arthritis (Schuler and Ehninger, 1995), the inflammatory and wasting effects of chronic tuberculosis (Klausner *et al*, 1996), Behcet’s disease (Hamuryudan *et al*, 1998), Crohn’s disease (Wettstein and Meagher, 1997; Ehrenpreis *et al*, 1999; Vasiliauskas *et al*, 1999) aphthous ulcers (Youle *et al*, 1989; Alexander and Wilcox, 1997; Jacobson *et al*, 1997), cachexia (wasting) associated with HIV infection (Sharpstone *et al*, 1995; Reyes-Teran *et al*, 1996) and AIDS-related Kaposi’s sarcoma (Fife *et al*, 1998). There is also a wide body of evidence from large-scale clinical trials showing the effectiveness of thalidomide as a treatment for refractory or relapsed multiple myeloma (MM) (Singhal *et al*, 1999; Hideshima *et al*, 2000; Juliusson *et al*, 2000; Kneller *et al*, 2000; Zomas *et al*, 2000), and this extends to the treatment of a number of other tumours (Eisen *et al*, 2000; Fine *et al*, 2000; Gutheil and Finucane, 2000; Patt *et al*, 2000; Tseng *et al*, 2001; Eisen, 2002).

Immunomodulatory drug analogues are able to costimulate T cells (Haslett *et al*, 1998; Corral *et al*, 1999) and are highly antiangiogenic (Dredge *et al*, 2002a). The ability to costimulate T cells has been associated with an increased Th1-type cytokine response and suggests that in certain clinical settings, IMiD analogues are likely to act as adjuvants to promote T-cell responses, thereby contributing to antitumour activity *in vivo*. In this regard, IMiDs have been shown to augment antitumour responses *in vivo*, leading to long-term protection from tumour challenge (Dredge *et al*, 2002b). Antiangiogenic activity has provided the rationale for the use of this class of compound as anticancer agents and, although unproven, this has often been linked to thalidomide's teratogenicity. However, the lead IMiD, CC-5013, has been shown to be both nonteratogenic and antiangiogenic in animal models.

The clinical development of the IMiDs has been initiated with the use of CC-5013 in MM (Marriott *et al*, 2001; Richardson *et al*, 2001; Zangari *et al*, 2001). This compound has recently completed a phase I clinical trials programme in which it was found to be safe and well tolerated. Furthermore, a report of clinical efficacy of CC-5013 in MM patients has recently been published (Richardson *et al*, 2002), although T-cell costimulation has not been demonstrated *in vivo*. We therefore conducted a phase 1 study of CC-5013 in patients with metastatic malignant melanoma with treatment administered according to an accelerated titration design with intrapatient dose escalation (modified from Simon *et al*, 1997). This disease has a median survival of 6–9 months and current systemic therapy can induce complete durable responses in only a small minority of patients (Atkins, 1997). Current chemotherapeutic options offer a very poor response rate of 14–20% and immunotherapy, for example interleukin (IL)-2 and interferon-*α* (IFN-*α*) have produced good responses in only a small number of patients.

In this study, we have assessed the safety, tolerability and clinical effects of CC-5013 during the treatment of 20 patients with metastatic malignant melanoma and other advanced cancers (Table 1

**Table 1:** Patient details: possible/probable CC-5013-related adverse effects

**Patient no.**	**Age (years)/sex**	**Disease**	**Stage^a^**	**Site**	**Previous treatment**	**Response^b^**	**Completed/withdrew**	**Days on study/maximum dose (mg)**	**Samples for *in vitro* analysis**
1	45/F	Malignant melanoma	IVabc	Right ileoinguinal area	Cancervax trial	PD	Completed	28/50	No
2	28/F	Malignant melanoma	IVabc	Right thigh	Dacarbazine (three cycles); peptides (melanA, gp100, tyrosinase) and IL-2	SD; partial response of liver	Completed	28/50	No
3	58/M	Malignant melanoma	IVabc	Lower abdomen	Dacarbazine; radiotherapy	PD	Progressed	14/10	No
4	44/F	Malignant melanoma	IVa	Abdomen	Adjuvant interferon	SD; minor response of abdominal nodes	Completed	28/50	No
5	16/M	Malignant melanoma	IVac	Right temporal region	Adjuvant interferon	PD; minor response of small cutaneous lesion	Completed	28/50	No
6	56/F	Malignant melanoma	IVabc	Vulva	Chemotherapy; bilateral groin radiotherapy	SD	Completed	28/50	No
7	31/M	Malignant melanoma	IVabc	Left upper chest	Debulking surgery; peptide (triple) and IL-2; dacarbazine; vindesine X3	SD; minor response of subcutaneous lesion	Completed	28/50	Yes
8	80/M	Malignant melanoma	IVab	Right side of neck	Treosulphan (six cycles)	PD	Completed	28/50	Yes
9	48/M	Malignant melanoma	IVa	Left foot	BCG (two doses)	SD; minor response of cutaneous nodules	Completed	28/50	Yes
10	56/F	Malignant melanoma	IVac	Breast	Vinflunine; radiotherapy	PD	Completed	28/50	No
11	23/M	Malignant melanoma	IVabc	Abdomen	Adjuvant interferon; radical radiotherapy	PD	Progressed	24/50	Yes
12	51/M	Malignant melanoma	IVac	Right thigh	Carboplatin, dacarbazine, vincristine, vinblastine & vindesine	SD	Completed	28/50	Yes
13	64/M	Malignant melanoma	IVabc	Right chest wall, right lower eyelid, lips	Multiple surgery only	PR, CT & complete subcutaneous response	Completed	28/50	Yes
14	52/M	Adenocarcinoma of the Pancreas	IV	Pancreas & portal vein	*M. vaccae*; retinoic acid & gemcitabine	PD; minor response serum CA19.9	Completed	28/50	Yes
15	47/M	Carcinoid	IV	Multiple carcinoid tumours	Radiotherapy; 5-fluorouracil & folinic acid; interferon; etoposide & cisplatin; thalidomide	N/E entered inappropriately	Withdrew	14/10	No
16	34/M	Adenocarcinoma of the Pancreas	IV	Head of pancreas	Gemcitabine & tomudex	PD	Completed	28/50	Yes (ELISA only)
17	55/F	Renal cell carcinoma	IV	Left kidney	Interferon; bryostatin; thalidomide; radiotherapy	N/E withdrew due to adverse effects	Withdrew	16/10	No
18	65/F	Ductal carcinoma of the breast	II	Breast	Mitozantrone, methotrexate & mitomycin; cyclophosphomide methotrexate & 5-fluorouracil; megace; 5-fluorouracil, epirubicin & cyclophosphomide; herceptin; tamoxifen; radiotherapy	N/E withdrew due to generalised weakness	Withdrew	14/10	No
19	66/M	Squamous cell carcinoma of the lung	IV	Right lung	Mitomycin, winblastine sulphate & cisplatin; radiotherapy	PD	Progressed	26/25	No
20	66/F	Adenocarcinoma of the lung	III	Left lung	Radiotherapy; mitomycin, vinblastine & cisplatin	PD	Completed	28/50	Yes

a *Staging*: a, skin, subcutaneous tissue, lymph node metastases; b, lung and gastrointestinal metastases; c, other sites including liver, bone and brain metastases.

b *Response*: PR=partial response; SD=static disease; PD=progressive disease; N/E=nonevaluable.

). We have also sought evidence of immunostimulatory and antiangiogenic effects by assessing multiple serological factors and changes in peripheral blood T-cell markers.

## MATERIALS AND METHODS

### Patient selection

Patients were recruited from a single oncology centre according to the following eligibility criteria: histologically or cytologically proven stage IV metastatic melanoma (*n*=13), unresectable adenocarcinoma of pancreas (*n*=2), renal cell carcinoma (*n*=1), ductal carcinoma of the breast (*n*=1), squamous cell carcinoma of the lung (*n*=1), carcinoid (*n*=1); age over 18 years; clinical or radiological evidence of disease progression in the 3 months prior to trial entry; life expectancy of over 2 months; adequate baseline organ function; and minimum body weight of 50 kg. Previous treatment with chemotherapy or radiotherapy was allowed with the exception of prior radiotherapy to the brain. For all patients, there was no known hypersensitivity to thalidomide or similar drugs and no previous anticancer therapy or experimental treatment for 30 days prior to study entry (for pancreatic patients, no concomitant chemotherapy). Women were excluded from the study if pregnant, lactating or not using adequate contraception. All patients gave written informed consent prior to participation in the study. The study protocol was approved by the local research ethics committee (LREC) and was conducted according to ICH good clinical practice and in accordance with the declaration of Helsinki.

### Treatment and assessment

CC-5013 was manufactured by Penn pharmaceuticals (Tredegar, Wales, UK) and supplied by Celgene Corporation (Warren, NJ, USA). Before initiating therapy, patients were subject to a complete medical history, physical examination and baseline evaluation of signs and symptoms, including full blood counts and a detailed neurological examination. These were repeated at weekly intervals during the study period and also at the end of the study. CC-5013 was taken in tablet form each evening and blood samples were collected at approximately the same time of the day at each visit. Therefore, analysis of serum cytokines and T-cell subsets was undertaken at a constant interval relative to the CC-5013 administration.

The treatment was administered according to accelerated titration design with intrapatient dose escalation (modified from Simon *et al*, 1997). All patients were treated with 5 mg day^−1^ CC-5013 for 1 week with the dose escalating to 10 mg day^−1^ at week 2, 25 mg day^−1^ at week 3 and 50 mg day^−1^ at week 4. The study treatment was terminated after 4 weeks; however, patients were allowed to continue on the final dose subject to tolerance and response. Treatment beyond 28 days was administered on a named patient basis and patients continued to be regularly assessed for toxicity and response.

The assessment of safety and tolerability of the study drug was based on the evaluations of the clinical laboratory tests, measurements of vital signs and the occurrence of adverse events during the study. The toxicity of the study drug was evaluated by means of the NCI-Common Toxicity Criteria (http://ctep.info.nih.gov/repo rting/ctc.html).

Evaluation of tumour response was performed following 4 weeks of treatment with CT scan and measurement of visible skin lesions. Response was classified as per WHO criteria.

### Analysis of serological factors

Blood was collected into serum separator tubes and left to clot for ∼30 min. Tubes were spun at 950 **g** for 10 min and serum was collected. Sera were frozen in aliquots at −70°C until assayed for sIL-2 receptor, IL-2, IL-12, tumour necrosis factor-*α* (TNF-*α*), IFN-*γ*, granulocyte–macrophage colony-stimulating factor (GM-CSF), vascular endothelial growth factor (VEGF), IL-8 and basic fibroblast growth factor (b-FGF) by ELISA. Standard absorbance (405 nm) of duplicate wells was used to calculate the concentration of cytokine/receptor levels.

### Phenotypic analysis of T cells

Heparinised venous blood was collected into sodium heparin vacutainers and surface stained (for 15 min at room temperature (RT)) with the following fluorochrome-conjugated monoclonal antibodies: anti-CD4 PerCP (Clone SK7; Becton Dickinson Immunocytometry Systems, BDIS, Oxford, UK) or anti-CD8 PerCP (SK1; BDIS) with anti-CD45RA FITC (L48;BDIS) and anti-CD45RO PE (UCHL-1;BDIS) plus appropriate isotype matched and compensation controls. Red blood cells were lysed with 2 ml 1 × FACS Lysing Solution (BDIS; 10 min, RT), samples were spun down (500 **g**, 5 min) and the cell pellet resuspended in 200 *μ*l CellFix (BDIS) for analysis. PBMC were surface stained (30 min, 4°C) with anti-CD4 or anti-CD8 PerCP FITC (SK11; BDIS) and anti-CD45RO PE plus appropriate isotype matched and compensation controls.

Upon flow cytometric analysis, lymphocytes were gated on forward scatter (FSC) *vs* side scatter (SSC) properties and PerCP-positive T-cell subsets were displayed as two-colour dotplots. For each sample, 10 000 lymphocytes were acquired on a Becton Dickinson FACScan using CellQuest™ software and analysed using EXPO32™ (Beckman Coulter).

### Statistical analysis

Serological and cell surface data comparisons between groups were examined by the Mann–Whitney *U*-test. The statistics were performed using GraphPad InStat 3 software.

## RESULTS

### Patient characteristics

The study recruited 20 patients, 13 with stage IV malignant melanoma, two with unresectable adenocarcinoma of the pancreas, one case of renal cell carcinoma, one of ductal carcinoma of the breast, one carcinoid and one patient with squamous cell carcinoma of the lung. (see Table 1 for patient details). In all, 12 patients were male and eight female. Age varied between 16 and 80 years with a median age of 51.5 years. The 16-year-old patient was recruited to the study following thorough discussion and agreement of the patient, his family and study investigators.

In total, 14 patients received the full 28 days of CC-5013 with no need for dose reduction. Three patients developed PD while on the study drug and did not complete treatment. One patient discontinued after 14 days, one after 24 days and one after 26 days. Three patients were nonevaluable due to withdrawal of consent to the study, two after 14 days and one after 16 days.

### Toxicity

Seven patients developed serious adverse events, none of them thought to be associated with the study drug. Patient 1 developed anaemia, requiring blood transfusion, as well as chest infection. Anaemia was most likely related to heavy bone marrow infiltration and disseminated disease. Following treatment of the infection, the patient completed the study. Patient 3 developed shortness of breath, which was due to accumulation of pleural effusion followed by chest infection and progressive disease. This patient was withdrawn from the study after 14 days of treatment. Patient 11 suffered dehydration and subsequent renal failure associated with poor performance status and disease progression. The patient was withdrawn from the study after 24 days. Patient 15 had prior spinal disease and was hospitalised due to spinal cord compression. The patient was withdrawn after 14 days and the overall impression was that the patient was entered inappropriately as he was not able to adhere to the study protocol. Patient 17 experienced a series of adverse events, mostly diarrhoea, abdominal pain and paraesthesia; none of those were more than grade 2, but he withdrew his consent to the study drug. Patient 19 was admitted to hospital in an acute confusional state, caused by a collapsed lung and increased dyspnoea. The patient was withdrawn from the study at day 26 and was admitted to a hospice. Patient 20 was hospitalised with worsened haemoptysis and dyspnoea but completed the study.

For 36% of the adverse effects, toxicity was classified as grade 1, 51% as grade 2, 8% as grade 3 and 5% as grade 4. There were several cases of grade 1 and 2 toxicity, some of them possibly associated with CC-5013 (see Table 2

**Table 2:** Adverse events reported during CC-5013 treatment

**Patient**	**Graded diagnosis**	**NCI toxicity grade**	**Causal relationship to REVIMID treatment**
2	Nausea and Vomiting	Grade 2	Remote
	Fatigue	Grade 2	Possible
	Altered taste	Grade 2	Possible
	Hot flushes	Grade 1	Possible
	Occasional cramps	Grade 1	Possible
4	Papular rash	Grade 1	Possible
6	Papular rash	Grade 2	Possible
8	Cramps	Grade 1	Possible
	Fatigue	Grade 2	Possible
13	Altered taste	Grade 1	Possible – although there is a local lesion
16	Jaundice	Grade 2	Remote
17	Numbness	Grade 2	Possible
18	Fatigue	Grade 2	Possible
20	Macular rash	Grade 1	Probable

). The most common were: paraesthesia, altered taste, papular, itchy rash, fatigue, nausea, poor appetite and vomiting. There was no evidence that somnolence is a problem for patients taking CC-5013. Apart from mild paraesthesia, there was no evidence of any neurological defects. Evidence of haematological toxicity included three patients who developed thrombocytopaenia and one who developed neutropaenia while on the study. There was no evidence for any biochemical toxicities in association with CC-5013 treatment.

### Evidence of clinical efficacy

Partial response (PR) was documented in one out of 20 patients (5%). Five patients had stable disease (SD) (25%) and 11 patients developed progressive disease (PD) (60%). There were no complete responses and three patients were not evaluable (patients 17 and 18 withdrew consent and patient 15 was entered inappropriately). Two of the patients developed a mixed response. Patient 2 had a decrease in the size of mediastinal mass and skin lesions but an increase in the size of liver deposit, and patient 14 had stable para-aortic lymphadenopathy but an increase in the number of lung and liver deposits.

**Figure 1:**
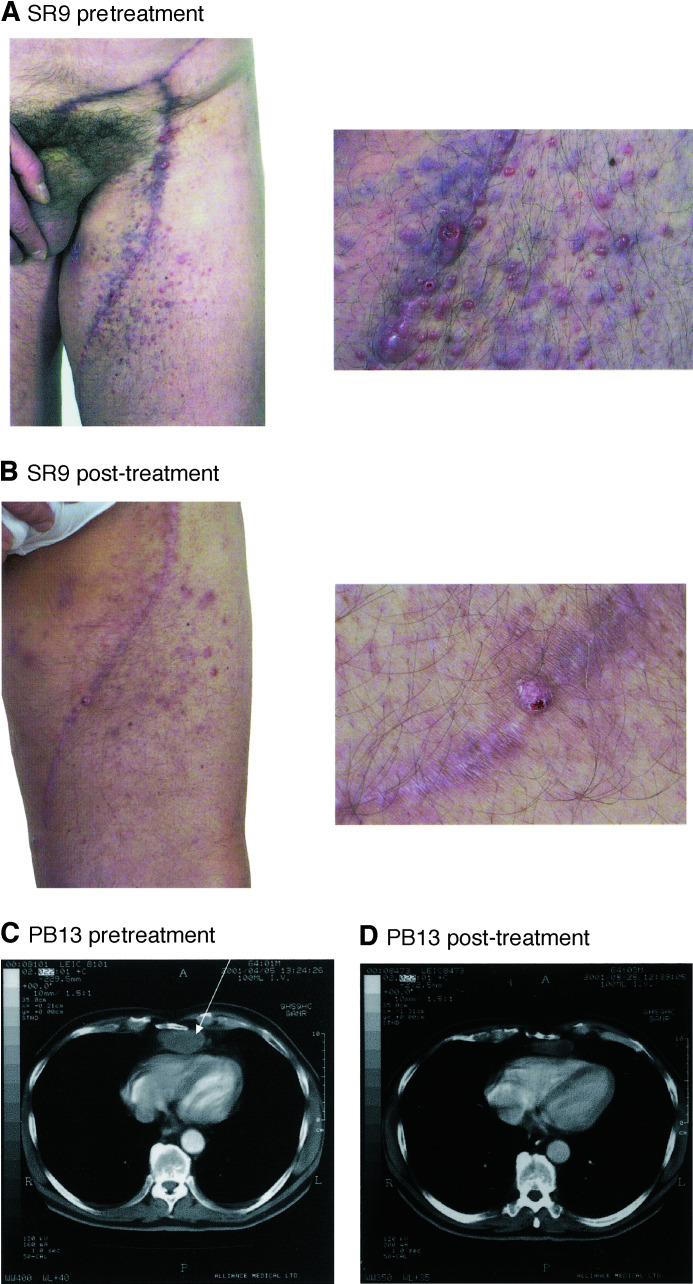
Evidence of clinical response during CC-5013 treatment. (**A**) Patient SR9 exhibited extensive nodular cutaneous disease over his left thigh. Following treatment (**B**) lesions became flattened and attenuated. (**C**) Patient PB13 shows mediastinal mass (arrowed) on CT scan which is reduced (**D**) after treatment.

Some patients developed minor responses that did not classify as PR but with a definite decrease in the size of visceral lymph nodes (patient 4), subcutaneous nodules (patient 7) and cutaneous disease (patient 9) (Figure 1

). In all, 10 patients continued CC-5013 at the final dose (50 mg) on a named patient basis with static disease and a good quality of life. These patients continued on CC-5013 for a mean of 3.5 months prior to disease progression. One patient continued on CC-5013 at 50 mg for 8 months during which time there was a minor response of pulmonary secondaries.

### Effect on immunologic and proangiogenic serum factors

**Figure 2:**
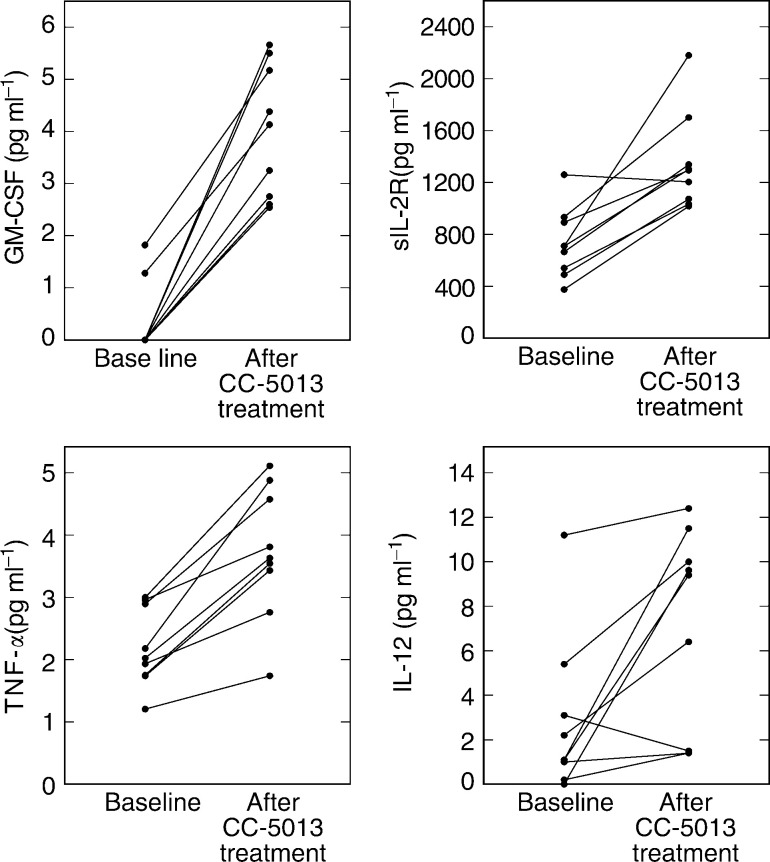
Changes in serum sIL-2 receptor, GM-CSF, TNF-*α* and IL-12 levels due to CC-5013 treatment. Levels pretreatment (baseline) are compared to levels at follow-up (at 4–5 weeks). sIL-2 receptor; ^*^P=*0.0005* (baseline *vs* follow-up). GM-CSF; ^*^P=<*0.0001*. TNF-*α*; ^*^P=*0.0056*. IL-12; ^*^P=*0.032*.

We detected increased levels of GM-CSF, TNF-*α* (both nine out of nine), sIL-2 receptor (eight out of nine) and IL-12 (seven out of nine) in the serum of patients after CC-5013 treatment compared to pretreatment levels (Figure 2

). Available serum samples from nine patients were assessed. Median sIL-2 receptor: baseline, 710 pg ml^−1^; follow-up, 1294 (^*^P=*0.0005*). Median GM-CSF: baseline, 0 pg ml^−1^; follow-up, 4.13 (^*^P=<*0.0001*). Median TNF-*α*: baseline, 2.02 pg ml^−1^; follow-up, 3.63 (^*^P=*0.0056*). Median IL-12: baseline, 1.1 pg ml^−1^; follow-up 9.4 (^*^P=*0.032*). We were unable to detect IL-2 or IFN-*γ* in any of the serum samples.

**Figure 3:**
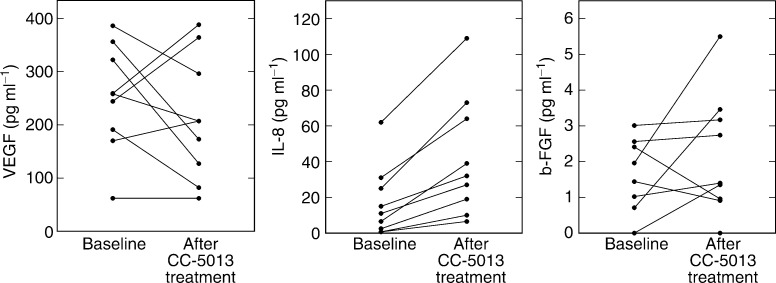
Detection of proangiogenic factors VEGF, IL-8 and b-FGF in the serum of patients is not significantly changed by CC-5013 treatment compared to baseline. VEGF; NS, *P*=*0.566*. IL-8; ^*^P=*0.047*. b-FGF; NS, *P*=*0.427*.

We also assayed serum to determine effects on the levels of proangiogenic factors (Figure 3

). Median VEGF: baseline, 258 pg ml^−1^; follow-up, 207 (NS, *P*=*0.566*). Median b-FGF: baseline, 1.44 ng ml^−1^; follow-up, 1.4 (NS, *P*=*0.427*). Median IL-8: baseline, 11 pg ml^−1^; follow-up, 32 (^*^P=*0.047*).

### Effect on peripheral blood T-cell CD45 isoform expression

**Figure 4:**
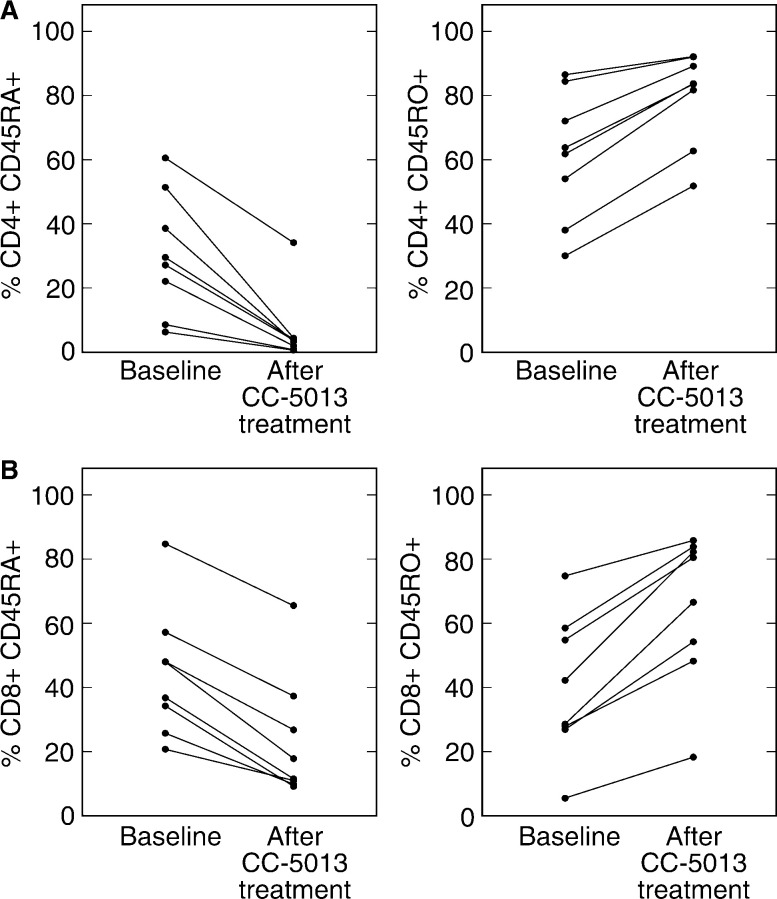
Effect of CC-5013 treatment on the CD4+ and CD8+ T-cell surface expression of CD45 isoforms. Data are expressed as percentage expression on cells’ pretreatment (baseline) and at follow-up (4–5 weeks after starting treatment). For CD4+ cells: CD45RA expression; ^*^P=*0.003*. CD45RO expression; NS, *P*=*0.105*. For CD8+ cells: CD45RA expression; ^*^P=*0.049*. CD45RO expression; NS, *P*=*0.065*.

We determined the expression of CD45 isoforms (CD45RA and CD45RO) on peripheral CD4+ and CD8+ T cells before CC-5013 treatment and at follow-up (at weeks 4 and 5). Figure 4

shows clear trends towards decreased CD45RA expression on both T-cell subsets in conjunction with increased expression of CD45RO. However, significance is only reached during analysis of decreased CD45RA expression on CD4+ cells. Median percentage CD45RA expression on CD4+ cells: baseline, 28.4%; follow-up, 3.5% (^*^P=*0.003*). Median CD45RO expression: baseline, 62.8%; follow-up, 83.6% (NS, *P*=*0.105)*. Similar results are seen with CD8+ cells. Median CD45RA expression: baseline, 42.4%; follow-up, 14.7% (^*^P=*0.049*). Median CD45RO expression: baseline, 35.4%; follow-up, 73.51% (NS, *P*=*0.065*).

## DISCUSSION

Disseminated malignant melanoma is a chemoresistant tumour with very poor prognosis. However, immunotherapy in advanced disease can offer durable responses and a greater understanding of the immune mechanisms involved may in the future offer better treatment for a wider group of patients. This study is the first to show the use of CC-5013 in patients with solid tumours, which include 13 patients with advanced melanoma. Despite the fact that clinical response was not the primary end point of the study and that the patients had not responded to prior therapy, we found clear evidence of drug activity in rapidly progressing malignant melanoma with one PR and two minor responses of visceral disease. Responses were noted in small volume cutaneous and subcutaneous disease. However, there was no correlation between clinical response and disease volume; the one PR occurred in a patient with relatively high volume visceral disease. It was also noted that patients subjectively felt better after commencing treatment. There was no therapeutic effect in other solid tumours treated, although three out of six patients were nonevaluable due to withdrawal or disease progression. Overall, it would appear that CC-5013 is capable of inducing antitumour activity at the doses tested and that static disease can be maintained even if only a minor response has been achieved.

Unfortunately, due to the small numbers of patients for whom immunological parameters could be measured, it was impossible to correlate the data with clinical response. However, we have found that irrespective of clinical response, there is strong evidence of immunological activation in all nine patients from whom samples were available for analysis. We assessed serum cytokine levels for evidence of immune activation. While serum levels of IL-2 and IFN-*γ* remained beneath the detection limit of the assays employed in the study, there were significant and consistently increased levels of sIL-2 receptor (in eight out of nine patients), providing evidence of T-cell activation. The strong induction of GM-CSF production by CC-5013 in all nine patients is further evidence of immune activation and is likely to lead to the stimulation and increased functional capacity of monocytes/macrophages and dendritic cells (Armitage, 1998), thereby potentially boosting the presentation of tumour antigens. The clinical use of recombinant GM-CSF as a cancer immunotherapy has led to reports demonstrating its benefits in the treatment of patients with melanoma (Armitage, 1998; Spitler *et al*, 2000).

The induction of GM-CSF may explain the significantly increased levels of TNF-*α* and IL-12 in these patients. These observations are consistent even in the serum of patients with rapidly progressing disease that did not respond clinically to CC-5013. Increased production of TNF-*α* and IL-12 by antigen-presenting cells is likely to drive Th1-type immune responses and enhance antitumour immunity. We have previously shown that the strong induction of TNF-*α* in *ex vivo* cultures correlates with a better prognosis in patients with colorectal cancer (Heriot *et al*, 2000).

Immunological activation was also assessed by the analysis of surface expression of CD45 isoforms on CD4+ and CD8+ T cells. The CD45RA+ T-cell phenotype represents nonactivated and mainly naïve cells. Upon activation, truncation of the CD45RA isoform exposes the CD45RO epitope. Our results show that there is a remarkably consistent and extensive shift in T-cell expression of CD45RA at baseline to CD45RO during treatment with CC-5013 (Figure 3). However, due to limited patient numbers, only the decrease in CD45RA expression reaches statistical significance. This apparent T-cell activation is evident on both CD4+ and CD8+ populations, supporting previous *in vitro* data showing that IMiDs are able to costimulate both T-cell subsets (Marriott *et al*, 2001). The total lymphocyte counts were generally increased in this patient group (data not shown), indicating that apoptosis of CD45RA+ cells is unlikely to account for the relative decrease in this population.

Our data suggest that CC-5013 is able to boost Th1-type cellular immunity and provide an environment for the generation of an antitumour response. Support for this concept has been obtained in a vaccination model of colorectal cancer in which another IMiD was shown to generate a protective and long-lasting antitumour response *in vivo* (Dredge *et al*, 2002b).

The serological changes observed in our study indicate that low-dose therapy is able to provide a significant immunological stimulus. In the context of tumour immunity, this may help to overcome the anergy associated with advanced cancer patients and boost cellular immune responses. It is worth noting that the progressive nature of disease in this patient group means that while immunological activation is seen in all patients, it is remarkable to also observe clinical responses and static disease in some patients. In this regard, it is possible that a correlation between immunological and clinical responses may become apparent during the treatment of patients with less advanced disease. The possibility that CC-5013 may boost previously induced immunity is suggested by the fact that five patients with minor responses and static disease had previously been given some form of immunotherapy, such as melanoma peptides or IL-2.

We have observed that CC-5013 is antiangiogenic using *in vitro* assay systems (Dredge *et al*, 2002a). Therefore, it is possible that this compound may also act *in vivo* to reduce the formation of new blood vessels, thereby inhibiting metastasis and preventing tumour growth. During our assessment of serum proangiogenic factors, we found that although levels of the neutrophil chemoattractant IL-8 were increased, VEGF and b-FGF were relatively unchanged. Interestingly, thalidomide has also been shown to have no effect on serum VEGF and b-FGF during the effective treatment of patients with multiple myeloma (Neben *et al*, 2001). Although other factors may also be important during angiogenesis, these results suggest that the T-cell costimulatory activity of CC-5013 (and perhaps thalidomide) may be of greater importance during the advanced cancer setting. Other evidence in MM patients suggests that natural killer (NK) cell activity may be important (Davies *et al*, 2001) and we have noted increased NK cell numbers in some patients after CC-5013 treatment (unpublished observation).

The possibility that CC-5013 can enhance the effect of prior or subsequent treatments cannot be ruled out. Prior immunotherapy, whether successful or not, may contribute to the immunostimulatory properties of CC-5013 and its antiangiogenic properties may enhance responses to radiotherapy and chemotherapy. Future studies will need to take these potential interactions into account. In conclusion, CC-5013 is safe, well tolerated and has some clinical benefit and immunological effects in the treatment of patients with refractory malignant melanoma and supports the continuing clinical development of this exciting class of compounds.
